# Leveraging governance strategies adopted by health facility governing committees in response to COVID-19 outbreak at the local level in Tanzania: A qualitative study

**DOI:** 10.1371/journal.pgph.0001222

**Published:** 2022-11-23

**Authors:** Anosisye Mwandulusya Kesale, Eliza Mwkasangula, Mikidadi Muhanga, Christopher Mahonge

**Affiliations:** 1 Department of Policy Planning and Management, College of Social Sciences and Humanities, Sokoine University of Agriculture, Morogoro, Tanzania; 2 Department Public Service and Human Resource Management, School of Public Administration and management, Mzumbe University, Morogoro, Tanzania; 3 Department of Development and Strategic Studies, College of Social Sciences and Humanities, Sokoine University of Agriculture, Morogoro, Tanzania; Makerere University, UGANDA

## Abstract

The governance of epidemics is very critical for curbing and responding to several infectious epidemics. This study was conducted to explore the experience of the Health Facility Governing Committees (HFGCs) on the governance strategies they adopted to levarage the COVID 19 epidemic in their primary health facilities in Tanzania. An exploratory qualitative design was employed to study the governance strategies adopted by HFGCs during the COVID19. In this study, fourteen (14) HFGC chairpersons and ninety one (91) HFGC members with experience regulating primary health centers during a COVID 19 pandemic were involved. The study included four (4) governance response metrics that were discovered to be commonly used by HFGCs. These included coordinating responders, providing health information, explaining health hazards, and conducting out health interventions. Despite variations in implementation strategies, only two (2) governance response measures, coordinating responders and implementing, were found to be consistently applied by the majority of HFGCs. The nature of the governance path chosen by the Tanzanian government has been found to have influenced the slow reaction of primary health care governance actors such as HFGCs. Despite being empowered by Direct Health Facility Financing, COVID 19 presented challenges to several HFGCs. Though observed to be autonomous and expected to make judgments based on their circumstances, higher-level governance actors’ opinions and actions on epidemics influenced the practices of local-level governance actors, including HFGCs. Indeed, for the HFGCs’ potential to be realized, they must be empowered in ways other than fiscal and political decentralization. Other aspects of empowering governance actors, such as capacity building and education level, should be considered in order for them to completely realize their potential.

## Introduction

Governance is considered to be the foundation for improving health service delivery at the primary health care (PHC) [1]. It refers to the process in which decisions are made and implemented to protect and promote population well-being [2, 3]. Governance is critical to achieving Universal Health Coverage (UHC). As a result, the government’s measures to improve governance at all levels promise better health outcomes and overall population well-being [4, 5]. Lower and middle-income countries (LMICs) have implemented a variety of measures to strengthen healthcare governance in primary care. Decentralization in various forms, including fiscal, administrative, and political decentralization, has been implemented to strengthen governance at primary health care facilities [5, 6]. Decentralization policy calls for the transfer of administrative, fiscal, and decision-making powers over health-care delivery to a subnational governing authority [7, 8]. Consistent with Alma Ata Declaration 1978 on the need for community participation in the governance and management of own health, decentralization has resulted into the establishment of Health Facility Governing Committees (HFGCs) to oversee health service delivery at the facility level [9]. These HFGCs are made up of local users/community members who are charged of planning and budgeting, ensuring availability of medicines and health commodities, procurement and linking community to health facilities [10]. Effective governance of basic health care is crucial for responding to complicated health shocks such as the COVID-19 outbreak. However, this can only be realized if there are empowered and well-performing HFGCs.

In the context of a health system, Pandemic/epidemic governance refers to the decisions and actions taken by governance actors to mitigate and respond to an outbreak for the greatest interest of the community [11, 12]. That is, strong and effective governance structures at all levels are critical for containing and responding to a wide range of infectious diseases. The epidemic puts all levels of governance, including primary health care, to the test, necessitating the adoption of new governance measures by governance institutions [13]. Epidemics are characterized by a lack of clarity in articulating problems, inconsistency in executing responses, and conflicting goals and cultures, necessitating the establishment of effective and innovative governance structures to address these crises. Traditional governance solutions, by definition, are no longer capable of addressing epidemic concerns. Empirical studies in primary health care suggest that in catastrophic situations, like COVID-19, governing actors must employ innovative and flexible tactics including developing networks, forming partnerships with stakeholders, mobilizing resources, and re-establishing new strategies. These are likely to protect health facilities, their employees, and the general public [1, 13–15].

In LMICs, governance of primary health care facilities is decentralized to HFGCs [16]. During an epidemic, health service users are delegated authority and responsibilities to monitor and improve service utilization, responsiveness, and provider accountability through [10, 17]. Epidemics, such as COVID-19, tends to sabotage and disrupt pre-determined healthcare plans and budgets due to its unique characteristics [18, 19]. To contain COVID-19-related issues, HFGCs must make multiple and timely decisions to improve facility operations and safeguard community health. Since the difficulties confronting COVID-19 governance are not purely technological, COVID-19 governance should deploy a mix of socio-political and cultural strategies, with the originality and innovation of HFGCs playing a critical role [15, 18, 20].

In Tanzania decentralized health care system, HFGCs were introduced in 1999 following the health sector reforms [21]. The HFGCs are composed of elected community members and health facilities in charge [9]. These HFGCs are assigned specific governance functions to perform such as participating in the planning, budgeting and procurement process. Also, mobilizing people to join community health funds, collecting, discussing and addressing community health challenges [22]. In terms of education, the HFGCs policy requires HFGC members to only be able to read and write in Kiswahili. Tanzania’s government has continued to implement several reforms to strengthen the health system, including empowering HFGCs to carry out their devolved functions. Tanzania’s current reform initiative is the implementation of Direct Health Facility Financing (DHFF). The Government of Tanzania (GoT) opted to implement Direct Health Facility Financing (DHFF) to ensure flexible and timely funding and utilization at the level of service delivery points in order to promote financial efficiency, accountability, and quality service delivery to the public [23, 24]. The current fiscal deistralization through DHFF to the primary health facility level is the extension of former fiscal decentralization that decentralized fiscal powers from the central government to the council level [25].

The World Health Organization (WHO) published procedures for dealing with outbreaks such as COVID 19 [26]. The framework outlines the critical four (iv) response tips that must be considered by management or governance actors to safeguard people from epidemics and other calamities. Indeed, the framework provides direction for health stakeholders in responding quickly to an outbreak. The established response measures are:-: (i) coordinating responders (ii) health Information (iii) communicating risks and, (iv) health interventions [33]. These guidelines establish a standard or framework on how health stakeholders, from the national level to primary health actors such as facility managers and governance actors, should respond to epidemics or outbreaks. In this context, it is believed that all decesions taken by HFGCs in response to epidemics should be based on WHO guidelines.

### COVID-19’S experience in Tanzania

The COVID-19 in Tanzania can be traced around March 2021, with the confirmation of the first case. Following this, Tanzania’s government banned all kinds public gatherings [27]. As part of the government’s further COVID-19 restrictions, all international passengers were required to spend 14 days quarantine in specific hotels. Indeed, the government made personal protective equipment (PPE) available to all levels of health care facilities [5]. Then, there was effective patient screening and isolation, quarantine of confirmed cases, and community mobilization to practice hand washing, sanitizing, and social distance. On May 8, 202, Tanzania’s then-president began proposing that traditional medicine, steam inhalation and ingesting indigenous herbs, be used to treat and prevent COVID-19 together with modern professional Covid-19 procedures [28]. The president and his administration encouraged everyone to pray in all houses of worship. Tanzania released statistics on Covid-19 cases and deaths early on (shortly after the first incidence), with full data set to be released on May 8, 2020. Tanzania has since halted the distribution of the Covid-19 surveillance report. The president claimed that the number of Covid-19 cases was declining significantly and thereafter declared herself Covid-19 free. As a result, Tanzanians were told to carry on with their everyday activities [27, 28]. Tanzania’s President refused to approve vaccinations even after other countries began to supply them. Other levels of government, such as ministries, regional and district levels, began to follow President Magufuli’s model of Covid-19 governance. The government had publicly questioned global health norms on testing and immunizations on several occasions [27, 28].

After the death of President Magufuli on March 17, 2021, the next president, Hon. Samia Suluhu Hassan, acknowledged the existence of Covid-19 in her inaugural address to the country and established an expert task committee to advise the government on the Covid-19 [27]. Following that, all Covid-19 protocols were required to be followed by all citizens, including all levels of government; as a result, testing resumed, and the Covid-19 surveillance report, which showed the number of cases and deaths, resumed to be released. The Ministry of Health, Community Development, Gender, Elderly, and Children (MoHCDGEC) developed and implemented Covid-19 standards to guide health care practitioners and other stakeholders in coping with epidemics. The vaccination was approved by the government, and President Samia Suluhu Hassan was among the first Tanzanians to be vaccinated, indicating that the Covid-19 vaccine is safe and effective. Tanzania is now implementing all Covid-19 processes, and residents are being immunized.

Several studies have been conducted to assess the response of Covid-19 management in Tanzania [27, 28]. The research undertaken has highlighted how Tanzania addressed Covid-19 at the national level and its effects from two presidential perspectives (President John Pombe Magufuli and Samia Suluhu Hassan). Indeed, Ruth et al. and Yamanis and Mollel research successfully identified Covid-19 containment measures that were adopted by Tanzania’s street-level bureaucracy and national government. However, studies have focused on bureaucracy levels while paying less attention to community governance structures such as HFGCs, which represent and link directly with communities and health facilities, more important are the final decision-makers at primary health care facilities. Therefore, is unknown how and what governance measures the empowered HFGCs in primary health care adopted to manage the COVID 19 epidemic in Tanzania. The goal of this study was to explore the governance measures implemented by HFGCs in Tanzania’s primary health care facilities.

## Materials and methods

### Study design

An exploratory qualitative design was employed to explore the governance strategies used by HFGCs during the COVID-19 outbreak. Considering that engaging the community through the HFGCs is a social process that is not linear, the chosen design was deemed appropriate. Since the COVID-19 outbreak was a new epidemic with unclear roles for HFGCs, a flexible design was required to establish the platform for future research by offering new insight into how HFGCs managed the outbreak within the WHO epidemic governance framework. A qualitative approach was used to assess epidemic governance at primary health care facilities in Tanzania’s Kilimanjaro and Songwe regions between February and April 2021. According to the Star Rating Assessment undertaken by the President’s Office-Regional Administration and Local Government of Tanzania in all primary health facilities in 2018, the Kilimanjaro region was picked as one of the regions with high health facility and HFGC performance [29]. Songwe was chosen to represent regions with low health facilities and HFGCs performance.

### Participants and recruitment

The study areas and respondents were chosen using a purposive sampling technique. Regions and councils were chosen based on their performance in the 2018-star rating assessment of all primary health care institutions in Tanzania. Songwe was chosen representing regions with low-performing health facilities and HFGCs., Kilimanjaro was chosen due to having good performing facilities and HFGCs. Then, from each region, two councils were chosen., Moshi Municipal was chosen in Kilimanjaro as a council with the lowest performing health facilities and HFGCs, as well as the urban local authority. Siha district council was chosen being the best performing council in the country according to the 2018-star rating evaluation, but it is also a rural local authority. In Songwe, Tunduma Town Council was chosen for being on its good performance in the region. Mbozi district council represented the region’s worst performer, but it is also a rural council, and Mbozi is the Songwe region’s headquarters. Two high-performing primary healthcare facilities and two low-performing primary healthcare facilities were chosen from each council. The health facilities were divided into two categories prior to the selection: health centers and dispensaries. As a result, a high-performing health center and dispensary were chosen from each council. A low-performing health center and dispensary were also chosen on purpose. Respondents were chosen from each of the health facilities. The respondents’ ability to offer meaningful information about the governance of health facilities during COVID-19 was one of the screening factors. As a result, all members of HFGCs were chosen for FGDs, and only the Chairpersons of the HFGCs of the primary health institutions that were chosen to implement DHFF qualified for interviews. A total of 14 in-depth interviews and 13 focus group discussions were conducted till the saturation threshold was reached. Saturation occurred when the participant began to respond in the same manner and no new information was provided. Each FGD consisted of 6 to 8 participants who are members of the HFGCs, Table 1 display the partcipants of the study.

**Table 1:** Characteristics of the study participants.

		HFGC members	HFGC chairperson	In Charges (HFGC secretariat)	Total No of Participants
Total Interviewed			14		14
Focus Group Participants		85		13	98
Age	≤ 35	31	4	7	
	≥ 36	54	10	6	
Sex					
Male		38	9	8	55
Female		47	5	5	57
Total Number of Participants					112

### Patients and public involvement

No patients were involved in this study.

### Data collection methods

The data was collected between February and April of 2021, during the COVID -19 outbreak, which included Tanzania. The study used in-depth interviews and Focus Group Discussions to collect data from respondents. FGDs were used to gather data from all HFGC members, while in-depth interviews were arranged with the HFGC chairpersons. The purpose of the interviews and focus groups was to have a better understanding of the governance methods implemented by the HFGCs during the COVID-19 epidemics. The respondents were asked what initiatives their HFGC as the health facility governance body had adopted to respond to the Covid-19, and how they had implemented those initiatives. Then, based on this major area of focus, sub-topics of what to discuss were determined to have a better understanding of how HFGC members dealt with the epidemic. Respondents were allowed to share their experiences with some probing questions to learn more about how they dealt with the COVID-19 outbreak, Table 2 show the manner in which interviews were conducted.

**Table 2:** Interview schedule.

No	Activity	Sub activity
1	Preamble	1. Interviewer introduction 2. Asking for consent from the participants and filling the consent form 3. Permission to record
2	Section A	1. What are the powers and responsibilities of your Health Facility Governing Committees? 2. What are the responsibilities of Health Facility Governance Committees in combating pandemics/epidemics
	Section B	1. As Health Facility Governing Committees what have you been doing/measures taken to contain Covid-19 2. How have you been accomplishing those measures? 3. What are the challenges your Health Facility Governing Committee is experiencing in the course of accomplishing its task?
	Section C	1. Do you think Direct Health Facility Financing has provided with any environment to better accomplish your responsibilities in containing Covid-19?

### Data analysis

The themes that arose during data collection were used to examine the data acquired for this study. The audio recordings of the in-depth interviews and focus groups were made. The audio recording was verbatim transcribed. Researchers were able to read and re-read the excerpts as a result of this. The textual extracts were then converted into codes based on the study’s focus areas, which were the governance measures used by HFGCs in the event of a COVID-19 epidemic. As a result, all specific areas in which respondents mentioned COVID-19 governance were coded. The created codes were used to capture significant themes related to governance techniques triggered by HFGCs during the COVID-19 epidemic, and the generated themes were linked to the study’s target area. All of the governance topics were examined and enriched from the acquired data to make them more significant and correspond with the study topic, and overlapping themes were merged into one. The themes were then fine-tuned and defined to ensure that the reader understood what they meant in the context of the COVID-19 epidemic.

### Ethical approval

The IRB with the number SUA/ADM/R. 1/8/668 was sought from the Sokoine University of Agriculture. The permit was then submitted to the President’s Office Regional Administration and Local Government (PO-RALG) to seek permit to research in the respective local government authorities. PO-RALG offered a permit with registration number AB.307/323/01 to allow the research to be conducted in the selected regions. Written Informed consent was obtained from all human participants of this study by completing the consent forms before they were involved in the study.

## Results

The collected data were organized into four governance response themes as suggested by WHO [30] which are responsible for coordinating responders, communicating risks, health information, and health interventions As a result, any subthemes that formed from the collection were guided to its main themes. In each theme, respondents were asked what they did or are doing in response to the Covid-19 in their jurisdiction.

### Coordinating responders

Epidemics like Covid-19 necessitate collaborative measures to contain and protect populations from all underlying threats. As a result, responsible local or governance structures, such as HFGCs, are needed to coordinate all parties to ensure that they work together to contain the epidemics. Regarding Covid-19, coordination of responders at the community or grassroots levels necessarily requires the identification of all stakeholders who will contribute to effective control and management of Covid-19 by HFGCs as governance actors responsible for governing service delivery at the facility levels. In doing so, the HFGCs should coordinate responders or stakeholders to develop a plan of action that outlines interventions aimed at controlling Covid-19, as well as specify the duties of each stakeholder.

The majority of respondents acknowledged that they organized responders in communities or villages by identifying them. Religious leaders, village government officials, business people, people with influence in the village, and NGOs or community-based organizations working in the village or given communities were among the stakeholders identified. Although the majority of respondents agreed that they identified certain interventions to be conducted by each stakeholder, many of them highlighted delivering education on social distancing and hand washing.

*"When we first heard about the disease*, *our HFGCs identified several key persons such as the church pastor*, *village chairperson and a man with a big shop in the village who could assist us in combating it*, *so we went to see them and talked to them*. …*we decided to meet with all stakeholders*.*"* Respondent-Mbozi District Council

Other respondents stated that the health facility in-charge assisted the HFGCs in understanding how to cope with the outbreak and that they discussed crucial stakeholders to be consulted with the chairperson. After that, they had HFGCs meeting to discuss what was going to happen at the stakeholder meeting. The majority of respondents claimed that healthcare personnel advised the HFGC on crucial matters and decisions that the committees needed to make.

*"As soon as we learned about the epidemic*, *we urged our HFGC chairperson to convene a meeting with facility personnel and HFGC*… *They then educated us on the epidemic and instructed us on what we should do during that meeting*. *We came up with new ideas that our HFGC was to implement such as sensitizing the community about covid-19 on washing hands and each household to have a washing hands facility at their doors"* Respondent-Siha District Council

Respondents believed that specific responsibilities to be assigned to stakeholders were not formally distributed, but that each stakeholder volunteered to do something that was within his or her capacity. However, those who are influential and have people such as religious leaders accepted sensitizing people on measures to respond to Covid-19 such as distancing and hand washing, which they also practiced in their worship houses. Other stakeholders, on the other hand, refused to do anything about Covid-19.

*"Of course*, *we specified activities for the stakeholders to carry out*, *but the main focus was on sensitizing communities to change their lifestyles*, *such as distance and hand washing*……*" We gave that duty to religious leaders because they are closer to the people"* Respondent-Moshi Municipal council

Other HFGCs in rural areas responded that reacting to Covid-19 was left to health workers and CHWs as it was a professional responsibility. They believed that governance matters such as approving the facility budget should be left to HFGC. As a result, it was left to the responsible health facility to determine who would assist in responding to the Covid-19. Others, believed that Covid-19 had been declared non-existent and that they had never seen it in their village.

*"I believe it is not within our mandates to meet with stakeholders*, *one respondent said during the focus groups*. *"Perhaps health workers can do that*… *We never got involved with that part here"* Respondent-Siha District Council

Another respondent said:-

*"We didn’t identify any stakeholder or arrange a meeting because we were told that the Covid-19 was just like any other disease and that only people who lived in the town were affected*,*"* Respondent- Mbozi District Council

### Health information

Monitoring information is critical in the setting of epidemics because it determines the impact of interventions implemented by health practitioners and governance actors. Throughout the crisis, practitioners and governance actors are crucial for directing decision-makers such as HFGCs. Surveillance and intervention information are critical for functional HFGCs during Covid-19. In this context, surveillance information entails providing a clear definition of the disease, HFGCs and service providers preparing to provide information to testing centers or laboratories, providing several cases, death statistics, and a map in a given community, sensitizing about the risk group, and elaborating on what has already worked to build community confidence.

Respondents gave varying responses on the roles they played concerning health information. While others stated that they were involved in providing health information to the community, specifically on awareness creation on Covid-19’s symptoms and informing community members on test sites for h Covid-19, others stated that they were never involved in doing so. Since the task was left to health workers when someone visited a health facility. Some claimed that, they lacked testing laboratories and instead assessed symptoms and referred patients to the council or a regional hospital. Some respondents stated that, despite providing information testing facility, the number of persons who attended was relatively low.

*"We’ve been educating people about the Covid-19 and trying to differentiate it from other diseases*… *But we’ve been doing it with CHWs or health-care workers but the turn up is very low*.*"* Respondent- Tunduma Town Council

Other said

*"We didn’t have any testing arrangements at our facility*, *and you know*, *Covid-19 doesn’t exist in our community because we’re poor*… *We’ve been told that the disease is only prevalent in urban areas”*. Respondent- Siha District Council

In terms of the number of cases and deaths, HFGC members stated that they have been updating communities about the cases and deaths through a variety of channels, including religious leaders, village leaders, and social media. The majority of respondents from rural settings, on the other hand, claimed that their HFGCs never disseminated information concerning cases and deaths because they either didn’t have any confirmed instances and deaths or were afraid of upsetting community members. Others believed that it was not their responsibility.

*"No one would dare to give statistics on cases and deaths since you need to double-check your data*… *Have you heard anything like that from the national level*? *So*, *how do we go about releasing it*?*"* Respondent-Moshi Municipal Council

#### Communicating risks

Like other epidemics, the number of cases and deaths tend to rise during the Covid-19 pandemic. During epidemics, significant increases in cases and deaths frequently result in the rapid spread of disinformation and misinformation, including rumors, gossip, and incorrect information. This makes epidemics like Covid-19 more complex, causing fear and confusion among populations. As a result, all responders, including HFGC, must have strategies in place to cope with an infodemic. Dealing with epidemics necessitate actors converting scientific material such as Covid-19 into lay language and format, establishing a stronger communication plan that provides relevant and trustworthy messages to the community, and having health monitoring measures to mitigate them.

The vast majority of respondents admitted to having no strategy for communicating risks to community members. During the Covid-19, respondents admitted that there was a surge of information from social media and other sources. According to one of the interviewees,

*"To be honest*, *our HFGC did nothing in terms of sharing Covid-19 information to the communities because we were short on information*, *and we assumed the in charge would come to better educate us and the people about this disease*. Respondent-Mbozi District Council

Some of them admitted that even HFGCs members, were confused about what was actually true about Covid-19, because even higher government figures appeared to hold opposing viewpoints on the issue, leaving HFGC members and communities in a dilemma.

*"It’s certainly frustrating; we don’t know whom to trust because everyone is saying different things about the same topics*, *and even our highest government officials are inconsistent on this*. *have you visited social media and seen what they are saying*?*"* Respondent-Tunduma Town Council

Few respondents from urban areas claimed that they had some strategies in place, such as using facility health care workers to deliver testing education and clarifying the message regarding the Covid-19 because of widespread misinformation on social media. majority of rural respondents believed that even health workers did not have clear information regarding Covid-19 in the early days, which surprised HFGCs because they rely on professional guidance.

*"For us*, *we decided that our tasks should include organizing meetings and visiting public places with health facility workers and CHW*, *as well as providing a space for this profession to explain what Covid-19 is all about because they are the ones who understand the disease best*.*"* Respondent-Moshi Municipal Council

Another response was

*"Even our health workers were unclear about the covid-19*, *particularly its symptoms*… *They were terrified*, *just like us*, *because they had not been taught or trained about the Covid-19"* Respondent-Mbozi District Council

### Health interventions

Covid-19, like other epidemics, demands intervention strategies to limit transmission, severe morbidity and mortality, and the impact on health-care system performance. As the governing body of the health facility, HFGCs were obliged to have established or taken a decision on interventions to address all of the issues identified. The Covid-19 was supposed to come out with interventions such as community engagement and promotion, case management and IPC. These had to include case isolation, early supportive treatment, and protecting health workers as the overseers of health service delivery and the organ that ensures whatever is done at the facility has their blessings. Other methods include testing, contact finding, contact tracing, vaccine promotion, and safe and dignified burials.

When HFGCs members were asked what type of health interventions were adopted in their communities or health facilities, their responses were diverse. Some respondents in rural areas, for example, stated that they conducted village meetings to give Covid-19 sensitization, where villagers were informed about how the disease is transmitted, measures to be taken by each village member, and where to go if one suspected contact with Covid-19.

*"We ensured that several HFGC members attended every village meeting*, *as well as the health facility in charge*, *to educate people about Covid-19*.…*sometimes we used even our local languages to help people understand properly"* Respondent-Mbozi District Council

While other responded:-

*"We devised a strategy to reach out to the community and raise knowledge about COVID-19*… *our members were then dispersed throughout the hamlet every Sunday to raise awareness through churches"* Respondent-Siha District Council

Respondents in urban regions stated that it was difficult to organize meetings due to the nature of urban life, thus it was essential to ensure that every household, and public institution, such as schools and offices, have washing hands gear and sanitizer. Other representatives stated that they lacked a community engagement and promotion strategy because the president announced that there would be no Covid-19 in Tanzania.

*"President Magufuli said that there was no Covid-19 in Tanzania*, *so how could someone go to a community and start telling people that it is Covid-19*?*" "We all relaxed and continue about our normal lives"* Respondent-Moshi Municipal Council

Regarding case isolation and early treatment, the majority of respondents stated that it was not implemented in their facilities since it was handled by higher-level facilities such as district and regional hospitals. Interestingly, some respondents in Kilimanjaro stated that they advised people to use traditional herbs and ginger and that it was working and helping them, even though this cannot be scientifically confirmed.

*"We’re utilizing traditional herbs here to protect ourselves from Covid-19*, *and it’s working well*… *The leaves were similar to those found in Madagascar*… *"Perhaps they boost our immunity"* Respondent-Tunduma Town Council

Regarding health worker protection, the majority of respondents stated that their governing bodies granted funding to purchase PPE, water tanks, masks, and sanitizer for their health facilities. They also required anyone who visited their health facilities to wear masks and wash or sanitize their hands before approaching any facility employee or office. They stated that all was doable due to the financial resources stored in their facilities.

*"In our HFGC*, *we took a budgetary decision to reallocate certain funds that were to be utilized for vaccination promotion to acquire some items that would aid to protect the facility employees*, *such as masks and water tanks"* Respondent- Moshi District Council

*"Because COVID-19 was not included in our facility plan*, *our HFGC has yet to make a funding decision*. *We’re also hesitant to plan anything monetary because we haven’t received funds from the government or other sources*, *such as health insurance"* Respondent- Mbozi District Council

*"We wrote letters and sent to stakeholders we thought may help us*… *The response was great*, *and as a result*, *we were able to obtain some quite valuable materials*" Respondent-Tunduma Town Council

However, several respondents from rural facilities stated that they were unable to obtain PPE due to a lack of funding, but they were able to purchase masks, sanitizers, and water tanks for the health facilities.

Respondents from urban HFGCs stated that it was difficult for them to perform contact tracing due to the nature of urban life, but those in rural areas stated that they were able to pursue contact tracing, particularly for those coming from urban areas. Showing stigma to persons arriving from urban regions, as well as the reception of Covid-19 in rural areas.

*"Whoever comes from the city*, *we have to follow up on her/his condition*, *and we have been warning people to be cautious with them because Covid-19 comes from cities"* Respondent-Siha District Council

Respondents had mixed feelings about safe and dignified burials. The majority said they did not participate directly in burial activities. These individuals citing fear of being exposed to disease as one of the reasons. Others said they did not participate because it was handled by health professionals at the district or regional level. Those involved in funeral events stated that they educate people about not participating fully in burial ceremonies, maintaining social distance, and wearing masks if they participate.

*"Because we don’t know the causes of many of the deaths that are happening at the time*, *we are asking community members not to participate*, *particularly when the death occurred far away from here and involves people from urban areas*… *Only a few relatives should attend"* Respondent- Siha District Council

## Discussion

Governance of epidemics such as COVID-19 is critical for limiting its effects on communities and health system performance in primary health care. HFGCs are the governance institutions overseeing community health service delivery. All important decisions on health service delivery are made by HFGCs, including how to respond to epidemics like Covid-19 in basic healthcare settings. The goal of this study was to explore the governance response strategies/mechanisms used by Tanzanian HFGCs to respond to the Covid-19 at the local level. HFGCs have had a mixed experience with the governance mechanisms implemented during the COVID-19. Some HFGCs were very active in using their devolved governance abilities and mandates to make decisions to respond to COVID-19, while others did not make the necessary governance decisions.

However, in the context of DHFF, this study demonstrates the common governance mechanisms employed by HFGCs in selected Tanzanian primary health care facilities. Governance techniques such as coordinating responses were utilized by HFGCs (identifying stakeholders, developing an action plan, and assigning responsibilities to each stakeholder). Furthermore, providing health information (directing them to testing laboratories, providing information on Covid-19 cases, deaths, and risk areas in their communities); communicating risks (using health facility staff and CHW to provide accurate information about Covid-19, as well as text messages to provide accurate information) (organized sensitization meetings, procured PPE, made the financial decision, contact tracing, implemented safe and dignified burial).

Participants reported that HFGCs were slow on responding to COVID-19 due unpreparedness for the outbreaks and hence did not know which approaches would be helpful in combating it. Lack of resources was also revealed to be a serious barrier to HFGC’s choice to adopt several HFGCs initiatives, such as the buying of PPE. This study supports the claims made in the literature that the healthcare system’s lack of preparedness during the rise of COVID-19 would hurt the epidemic’s combat [31–33]. Despite the difficulties, HFGCs played a significant role in guaranteeing the availability of basic healthcare supplies such as PPE, masks, and sanitizers. Governance approaches helped to fill the budget gap in primary health facilities by mobilizing stakeholders and resources to obtain medical commodities and medicines that were required. This also corresponds to McMullin and Raggo [34] who stated the importance of collective efforts among health stakeholders in dealing with the COVID 19.

The Covid-19 experience in Tanzania, specifically from May 2020 to March 2021, has been found to contribute to a variety of government interventions to epidemics. During this time, the government chose its own approach rather than following the worldwide Covid-19 standard protocol, with the president routinely opposing protocols, forcing other senior government officials to follow suit. This confused community/local government actors, who were hesitant to reply to the Covid-19 in accordance with professional protocol. Furthermore, even when governance actors chose to initiate Covid-19 initiatives in order to comply with Covid-19, they were divided or rejected by community members who used the president’s Covid-19 statements to explain their rejections. To a large extent, the governance systems adopted at the national level have determined government at the local level in Tanzania.

There is little or no difference in the roles given to HFGCs in Tanzania based on the tactics used in combating Covid-19 [35]. Many initiatives remained within their scope of authority and functions, although they were to be implemented in circumstances that were uncommon for HFGCs. According to the guideline [35] In Tanzania, HFGCs are responsible for planning, procurement, budgeting, health promotion, connecting community and health facilities, supervising facility infrastructure construction and maintenance, and overseeing facility assets. This raises some concerns when respondents claim to have done nothing or were unaware that dealing with epidemics was part of their responsibilities. The members of the HFGCs’ educational level may have led to the majority of them being unaware and failing to make choices within their mandates. According to the HFGC rules, members of the HFGCs are simply required to know to read and write swahili language. Given the authority and authority vested upon HFGCs, we believe that their members require education beyond reading and writing swahili in order to carry out their duties efficiently.

HFGCs in urban councils were found to be more active in dealing with Covid-19 than HFGCs in rural councils. The majority of urban HFGC respondents agreed that they made some decisions to respond to the Covid-19, but the majority of rural HFGC respondents said that they made no or few decisions to react to the Covid-19. Urban HFGCs made the decision to organize stakeholders, mobilize stakeholder resources, provide health information such as testing laboratories and isolation centers, carry out actions such as suspect isolation, and protect health workers by purchasing PPE. On the other hand, rural HFGCs decided on community meetings, contact tracing, procuring masks, sanitizers, and water tanks, and deploying health professionals and CHW to conduct Covid-19 education. The variance in the strategies implemented by these HFGCs could be attributed to the nature of the Covid-19 epidemics in Tanzania, which were more widespread in urban than in rural areas. As a result, the demand for responses was significantly higher in urban areas than in rural areas. However, no significant difference in Covid-19 governance in Tanzania was found between HFGCs with high and low performance in the star rating assessment completed in 2018. In dealing with Covid-19, both high and low HFGC made roughly similar decisions. The location of the HFGCs was important in deciding the governance mechanisms implemented by a specific HFGC.

Direct Health Facility Financing (DHFF) was reported to have enabled HFGCs in making decisions about planning, procurement, and budget allocation to some operations in the context of controlling the Covid-19 outbreak in Tanzania. Prior to DHFF, HFGCS and health facilities could not make those decisions without district-level approval, or they may develop plans but fail to spend financial resources to carry them out. As money were maintained in council accounts rather than facility accounts, this is the case. During the pandemic, however, it was discovered that the majority of HFGCs in both urban and rural regions were unaware of their powers and obligations during epidemics. This implies that, even though the DHFF permits HFGCs to freely make decisions about planning, procurement, budgeting, and financial management, members are unaware of how to execute such powers. This means that the government’s goals of increasing community control in primary health care delivery may not be met. As a result, empowerment entails more than just budgetary decentralization, and more efforts are required to improve individual ability among members of the HFGCs.

HFGCs have been found to partially comply with the WHO framework for managing epidemics. The framework emphasizes Coordinating Responders, Health Information, Communicating Risks, and Health Intervention as critical tips for managing or responding to epidemics such as Covi-19. However, the majority of HFGCs in the study were found to focus on two recommendations, coordinating responders and delivering interventions, but little on health information and communicating risks. Failure to implement health information and communicate risks jeopardizes the effectiveness of HFGCs in implementing health intervention because the two components shape intervention implementation for the actors. Indeed, the responses of HFGCs to the four managing epidemics tips are shaped by the health service providers/staff of the specific HFGCs because they are the ones that advise the HFGCs on critical technical issues such as Covid-19. Because HFGC members are not health professionals, where the health facility in-charge/staff has a good relationship with HFGC, the function tends to be good, and where the relationship is not strong, the HFGC is very limited.

## Conclusion

Effective epidemics governance is critical for limiting its effects at the community level. empowered governance institutions such as HFGCs have the promise for the achievement of effective epidemics governance. While there is evidence of some governance activities being pursued by HFGCs as governance actors in responding to Covid-19 in primary health care, higher-level governance actors may influence their practices to be effective or in effective. Indeed, for the potential of the HFGCs to be realized, the empowerment of HFGCs should not be centered on one component such as fiscal decentralization. Other aspects which involve empowerment of governance actors such as building the capacity of HFGCs members and re-thinking on the education level of the governance actors such as HFGCs need to be settled to fully realize their potential. In times of COVID-19, the functionality of HFGCs in low and middle-income nations appears promising, but more work is needed to unlock their potential and adequately respond to the epidemics.
